# Comparative Evaluation of Amoxicillin–Clavulanate and Ertapenem in an Exploratory Rat Model of ESBL *E. coli* Peritonitis

**DOI:** 10.3390/biomedicines14030500

**Published:** 2026-02-25

**Authors:** Elie Haddad, Bassem Habr, Hussein Nassereddine, Nassim Fares

**Affiliations:** 1 Faculty of Medicine, Department of Infectious Diseases, Hotel Dieu de France Hospital, Saint Joseph University of Beirut, Beirut 1107 2020, Lebanon; elie.haddad8@usj.edu.lb; 2 Faculty of Medicine, Department of Pulmonary and Critical Care, Hotel Dieu de France Hospital, Saint Joseph University of Beirut, Beirut 1107 2020, Lebanon; bassem.habr@usj.edu.lb; 3 Faculty of Medicine, Department of Pathology, Hotel Dieu de France Hospital, Saint Joseph University of Beirut, Beirut 1107 2020, Lebanon; hussein.nassereddine@hdf.usj.edu.lb; 4 Laboratory of Research in Physiology and Pathophysiology, Faculty of Medicine, Saint Joseph University of Beirut, Beirut 1107 2020, Lebanon

**Keywords:** inflammation, infection, *E. coli*, ESBL, peritonitis, cytokines, murine model, amoxicillin–clavulanic acid, ertapenem

## Abstract

**Background**: Extended-spectrum β-lactamase (ESBL)–producing *Escherichia coli* represent an increasing therapeutic challenge. While ertapenem (ERP) is commonly used as first-line therapy, amoxicillin–clavulanic acid (AMC) achieves therapeutic concentrations in serum and ascitic fluid and may offer a narrower-spectrum alternative. This exploratory preclinical study evaluated whether AMC produces effects comparable to ERP in a rat model of ESBL *E. coli* peritonitis. **Methods**: Thirty-three male rats were allocated to four groups: untreated *E. coli*, AMC, ERP, and sham controls. Peritonitis was induced by intraperitoneal injection of an ESBL-producing *E. coli* strain. The primary outcome was peritoneal bacterial culture positivity. Secondary outcomes included plasma inflammatory cytokines (CRP, PCT, TNF-α, IL-1β, IL-6), proteomic signaling markers, and histopathological inflammation scores in the peritoneum, spleen, and lungs. **Results**: One death occurred in the untreated group. Both AMC and ERP were associated with lower peritoneal culture positivity compared with untreated animals, with ERP achieving statistical significance and AMC showing a similar downward trend. Inflammatory cytokines and proteomic markers demonstrated comparable reductions in both treated groups. Histopathology showed reduced inflammatory scores, with AMC exhibiting the lowest peritoneal inflammation. Lung involvement was observed in 7/10 untreated rats versus 5/10 AMC- and ERP-treated rats; these differences were not statistically significant, reflecting the limited sample size. **Conclusions**: AMC and ERP produced broadly comparable effects on microbiological, biochemical, and inflammatory parameters in ESBL *E. coli* peritonitis. These findings suggest that AMC may merit further investigation as a potential narrower-spectrum option, though definitive comparisons require larger, adequately powered studies.

## 1. Introduction

Antimicrobial resistance (AMR) is one of the preeminent threats to global public health [1]. Over recent decades, the prevalence of multidrug-resistant (MDR) bacteria has surged, prompting the World Health Organization (WHO) to rank AMR among the top ten global health threats. These infections are increasingly common in both healthcare and community settings [2], often leading to poorer clinical outcomes. Such prognoses are typically driven by delays in appropriate antibiotic initiation and the subsequent reliance on alternative therapies, which are frequently less effective or carry higher toxicity profiles [3].

Of particular concern is the rising prevalence of bacteria producing extended-spectrum beta-lactamases (ESBL) [4]. These enzymes hydrolyze common antibiotics like penicillin and cephalosporins, rendering them ineffective. ESBL-producing Enterobacteriaceae frequently infect otherwise healthy individuals, and their carriage rate in the general population is steadily climbing [5]. Specifically, the *E. coli* sequence type 131 (ST131) clone emerged globally in 2008; it has since become the dominant extraintestinal pathogenic *E. coli* clone responsible for MDR worldwide [6].

Spontaneous bacterial peritonitis (SBP)—an infection of ascitic fluid lacking a surgically treatable intra-abdominal source—remains a frequent complication for patients with cirrhosis and ascites [7]. Per the IDSA 2024 Guidance on Antimicrobial-Resistant Gram-Negative Infections [8], effective management is critical. Amoxicillin-clavulanic acid (AMC) achieves therapeutic concentrations in both serum and ascitic fluid following intravenous administration [9]. In a non-comparative study, Grangi et al. demonstrated that AMC was highly effective, resolving 85% of 27 SBP episodes without significant adverse effects [9]. Similarly, clinical success rates for ertapenem (ERP) have reached approximately 84% [10].

To investigate antibiotic efficacy in peritoneal infections, various animal models are employed, including cecal ligation [4] and direct inoculation models where a syringe is used to implant an inoculum into the peritoneal cavity to induce infectious peritonitis [11].

Our aim was to determine whether AMC produces effects comparable to ERP across predefined outcomes—microbiological (primary: peritoneal culture positivity), inflammatory cytokines, proteomic signaling, and histopathology—in this acute ESBL *E. coli* peritonitis model. The study was exploratory and hypothesis-generating; it was not powered to establish equivalence or non-inferiority in a murine model; by investigating the comparable effect of AMC compared to ERP in the treatment of ESBL *E. coli* peritonitis. However, the present study does not model cirrhosis-associated SBP. Rather than spontaneous infection arising from portal hypertension, altered intestinal permeability, ascites, and immune dysfunction typical of cirrhosis, our work employs an acute, experimentally induced ESBL *E. coli* peritonitis model in otherwise healthy rats. Acute induction model should not be over-extrapolated to human SBP without further corroboration in disease-relevant models.

## 2. Materials and Methods

### 2.1. Ethical Statement

All procedures were approved by the Saint-Joseph University Ethical Committee (USJ-2024-25) and followed the American Physiological Society’s guiding principles, the NIH Guide for the Care and Use of Laboratory Animals, and EU Directive 2010/63/EU. The animals were housed in a controlled specific pathogen-free environment in individually ventilated cages with hygienic bedding from crushed beech wood (EAN5410340230947; Versele-Laga, Deinze, Belgium) and were exposed to a constant temperature of 25 °C and humidity level of 50 ± 5%, and a 12:12 h light/dark cycle with free access to food and water.

### 2.2. Sample Size Justification

In accordance with the ARRIVE 2.0 Essential Item 2, this study used 33 rats allocated into four groups (ERP *n* = 10; AMC *n* = 10; *E. coli* untreated *n* = 10; Sham *n* = 3). The experimental unit was the individual rat. No formal a priori power calculation was performed. As this was an exploratory pilot study designed to characterize microbiological, inflammatory, proteomic, and histopathological patterns, the use of approximately 10 rats per group follows common practice in exploratory peritonitis/sepsis models and allows estimation of variability while adhering to the principle of using the minimum number of animals required for meaningful results, in line with ARRIVE 2.0’s emphasis on justified sample-size decisions and ethical use of animals. The selected group size was therefore appropriate for detecting directional trends across multiple biological endpoints and for informing future adequately powered hypothesis-driven studies. To limit animal use in accordance with ethical guidelines, the Sham group was intentionally restricted to *n* = 3, serving only as a baseline reference rather than a fully powered comparator group.

### 2.3. Assessment and Data Analysis

The primary outcome was peritoneal bacterial culture positivity, supplemented by secondary measures including plasma cytokines (CRP, PCT, TNF-α, IL-1B, IL-6), histopathological inflammation scores of the peritoneum, spleen, and lung, and proteomic markers (NF-kB, NFATC3, ERK1/2, caspase-3). To ensure objectivity, histopathology scoring, proteomic quantification, and statistical analyses were performed by blinded assessors. Statistical evaluations were conducted using GraphPad Prism 9, employing Shapiro–Wilk tests for normality followed by one-way ANOVA with Sidak post hoc or Kruskal–Wallis with Dunn’s tests as appropriate. At the conclusion of the 8-day schedule, rats were euthanized via ketamine/xylazine anesthesia followed by bilateral thoracotomy and exsanguination. All results are interpreted as exploratory, focusing on the comparable effects of AMC and ERP to inform future powered studies. Investigators administering inoculations and treatments were not blinded to group identity due to logistical constraints, but all outcome assessments involving histology and protein quantification were performed under blinded conditions.

### 2.4. Murine Peritonitis/Sepsis Model

The ESBL-producing *E. coli* strain was obtained from the Saint-Joseph University microbiology laboratory. Identification was confirmed by Gram stain, API 20E bioMérieux (Analytical Profile Index), and mass spectrometry (MALDI-TOF). Susceptibility testing used disc diffusion (Kirby–Bauer method) with interpretive criteria in accordance with EUCAST recommendations [12].

On Day 1, rats received an intraperitoneal inoculum (≈1.5 × 10^8^ *E. coli* in 1 mL saline) combined with 125 mg talc to prevent spontaneous resolution of the infection and sustain peritoneal inflammation [13]; The turbidity was adjusted to a 0.5 McFarland standard prior to inoculation. Antibiotic treatment began 2 h after inoculation and continued according to each regimen’s schedule repeated over the following hours depending on the specific pharmacokinetic properties of each antibiotic.

### 2.5. Pharmacokinetic Rationale

The thirty-three rats were divided into four groups: ERP group (*n* = 10); Ertapenem administered subcutaneously at a dose of 150 mg/kg per day [14]; amoxicillin/clavulanic acid administered subcutaneously at a dose of 100 mg/kg per day every 12 h [15]; *E. coli* group (*n* = 10); Sham group (*n* = 3); rats received a 1 mL intraperitoneal injection of saline.

The ERP dose of 150 mg/kg/day [16] SC is well established in rodent studies, where β-lactams typically require 40–70% of the unbound fraction of the drug (fT > MIC) to produce bactericidal activity. Thus, 150 mg/kg/day SC represents a literature-supported dose likely to achieve therapeutically relevant fT > MIC in rats and is commonly used in rodent infection models, providing an appropriate comparator for AMC in this exploratory study. The AMC dose of 100 mg/kg every 12 h is consistent with veterinary and laboratory-animal dosing guides for β-lactam antibiotics in small animals and rodents [17].

The duration of the experimental protocol is defined as 7 days. Animal euthanasia was performed on day 8.

### 2.6. Animal Euthanasia

The euthanasia of rats in this study was performed in accordance with the most recent AVMA (American Veterinary Medical Association) Guidelines for the Euthanasia of Animals 2020 Edition. On Day 8, animals were anesthetized with intraperitoneal ketamine (100 mg/kg^−1^; Panpharma, Luitré, France) and xylazine (10 mg/kg^−1^; Interchemie, Waalre, Holland) to ensure rapid and painless unconsciousness. After confirmation of deep anesthesia, by the absence of pedal withdrawal reflex, the animals were euthanized by bilateral thoracotomy and exsanguination through heart excision to ensure a quick and humane death. Blood was collected into EDTA tubes and centrifuged at 4500 rpm for 10 min. The collected plasma was stored at −80 °C. In addition, peritoneal swabs and spleen tissue were obtained for culture. A portion of the spleen was crushed and inoculated onto MacConkey agar medium. The samples were incubated at +37 °C.

### 2.7. Microbiological Culture Assessment

Peritoneal and splenic bacterial burden was assessed with a binary scoring system: 0 = no growth (no colonies detected) and 1 = growth (any detectable colonies). Cultures were therefore semi-quantitative (presence/absence) rather than Colony Forming Units (CFU)-based counts, reflecting the small-volume constraints of this model and the directional objective of the study.

### 2.8. Plasma Concentration Measurements of CRP, Procalcitonin, TNF-α, IL-1β, and IL-6

Plasma concentrations of CRP, Procalcitonin, TNF-α, IL-1β, and IL-6 were determined by ELISA, an immunoassay technique primarily used in immunology to detect and/or quantify a protein in biological fluid. This technique falls within the broader framework of enzyme-linked immunosorbent assays, in which the assay is coupled with a catalytic reaction by an enzyme that releases a colored component followed by spectroscopy. This biochemical test uses one or two antibodies. The first antibody is specific to the antigen, while the other, coupled with an enzyme, reacts with immune complexes (antigen–antibody). For CRP, procalcitonin, TNF-α, IL-1β, and IL-6 assays, the kits used are specific for rats and are obtained from Abcam, UK. The kits are: ab46070, ab100767, ab119548, ab100777, and ab108827, respectively. For procalcitonin, the rat-specific CSB-E13419r kit from CUSABIO, Houston, TX, USA, was used.

### 2.9. Antibody Array

Antibody array was performed on three randomly selected samples from each of the Sham, *E. coli*, AMC, and ERP groups. Spleen and peritoneal tissues were homogenized and lysed in RIPA assay lysis buffer including protease and phosphatase inhibitors. Protein concentrations were measured using the Bradford protein assay (Bio-Rad, Marnes-la-Coquette, France), and samples were denatured in Laemmli loading buffer (Bio-Rad, Marnes-la-Coquette, France) containing 10% β-mercaptoethanol (Sigma-Aldrich, St. Louis, MO, USA) at 37 °C for 20 min. Proteins were separated by SDS 12% PAGE and transferred to polyvinylidene fluoride membranes (Bio-Rad, Marnes-la-Coquette, France) blocked with 5% bovine serum albumin. The membranes were incubated overnight at 4 °C with the corresponding primary antibodies: Anti ERK1/2 (1/1000)—Abcam ab17942, Anti p-ERK (1/1000)—Abcam ab78238, Anti NFkB (1/1000)—Abcam ab16502, Anti p-NFkB (1/1000)—Abcam ab86299, Anti NFATC3 (1/1000)—Santa Cruz sc-8321, Anti p-NFATC3 (1/1000)—ab59204, Anti Caspase 3 (1/1000)—Abcam ab13847. Blots were re-probed for glyceraldehyde-3-phosphate dehydrogenase (GAPDH) (5174; 1/1000) from Cell Signaling Technology, Danvers, MA, USA.

Goat anti-rabbit (1/3000, Bio-Rad Laboratories, Marnes-la-Coquette, France) was used as the secondary antibody. An imaging system fitted with a CCD camera was used to detect enhanced chemiluminescence signals (Omega Lum G, Aplegen, Gel Company, San Francisco, CA, USA). Image Studio Lite Ver 5.2 (LI-COR Biosciences, Lincoln, NE, USA) was used to do the quantifications. To generate antibody arrays, 5 µg of proteins were immobilized onto PVDF membranes using dot blot apparatus (Cleaver Scientific, Rugby, UK) before blocking and incubating with the primary antibody.

### 2.10. Proteomic Quantification Workflow

Peritoneal and splenic protein expression was quantified using a standardized workflow integrating dot-blot densitometry and Western blot analysis. Tissue lysates were prepared in RIPA buffer containing protease and phosphatase inhibitors, with total protein concentrations determined via the Bradford assay. For dot-blotting, 5 μg of protein per sample were immobilized on PVDF membranes; for Western blotting, equal protein amounts were separated by SDS-PAGE before transfer. Membranes were blocked and incubated with primary antibodies targeting NF-κB, phospho-NF-κB, NFATC3, phospho-NFATC3, ERK1/2, phospho-ERK1/2, and caspase-3. Following secondary antibody incubation, chemiluminescent signals were captured using a CCD imager and quantified via densitometry using Image Studio Lite. To ensure analytical rigor, a dual-normalization strategy was employed: phosphorylated proteins were expressed as a ratio of their respective total protein levels (e.g., p-NF-κB/NF-κB), while total protein expression levels were normalized to the housekeeping protein GAPDH (e.g., Caspase-3/GAPDH). All data were expressed as relative densitometric units scaled to the mean of the Sham group. Consequently, all figure axes are annotated to reflect these specific normalization methods, such as “p-ERK/ERK (normalized expression)” or “Caspase-3/GAPDH (relative units)”, to facilitate precise interpretation.

### 2.11. Histological Assessment

Histological examinations were conducted by two independent pathologists. Formalin-fixed, paraffin-embedded peritoneum, spleen, and lung sections were stained with hematoxylin–eosin. After staining, the sections were rinsed with distilled water, dehydrated in ethanol/water baths with a progressive decrease in water content, and finally rinsed in xylene before being placed in permanent mounting medium. Inflammation was graded on a 0–4 ordinal scale (none, minimal, mild, moderate, severe) by two pathologists under blinded conditions.

### 2.12. Statistical Analysis

Statistical data were analyzed and are presented as the mean ± standard error of the mean (SEM). Sample sizes (N) for each experiment are specified in the corresponding figure legends. Data normality was assessed using the Shapiro–Wilk test. For multi-group comparisons, we employed ordinary one-way ANOVA for normally distributed data, with Sidak’s post hoc test used for pairwise comparisons. Non-normally distributed data were analyzed via the Kruskal–Wallis test followed by Dunn’s multiple-comparisons test. Statistical significance was defined as *p* < 0.05.

## 3. Results

### 3.1. Body Weight Modification

The normalization of rats’ body weight between D1 and D8 showed no significant difference between all groups. In addition, regarding the mortality rates, only one rat died on the 4th day in the *E. coli* group.

### 3.2. Effects of Antibiotics Results on Positive Culture

**Figure 1:**
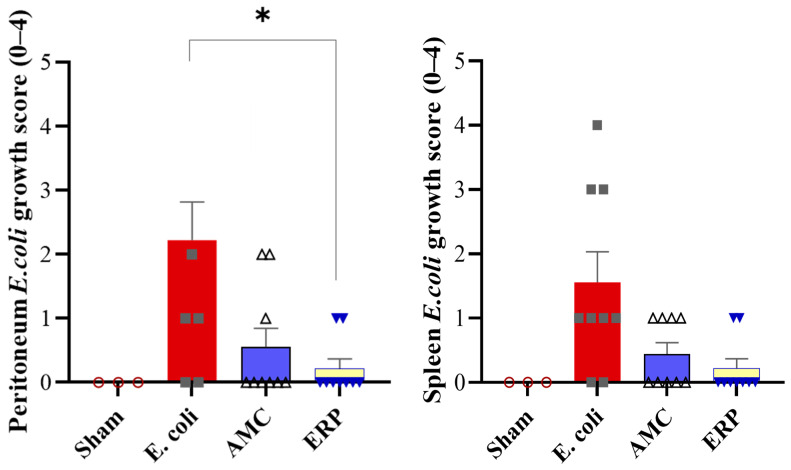
Peritoneal and spleen *E. coli* cultures: on the left peritoneal cultures and on the right spleen cultures. * *p* < 0.05.

In the peritoneal cultures (Figure 1), the mean microbiological positivity was significantly lower in the ERP group (0.1 ± 0.32) compared to the *E. coli* group (0.5 ± 0.53; *p* < 0.05). The AMC group also showed a downward trend (0.2 ± 0.42), though this did not reach statistical significance (*p* = 0.063). For the spleen cultures (Figure 1), no statistically significant differences were observed between groups. However, a consistent numerical decrease was noted in both the AMC (0.1 ± 0.3) and ERP (0.1 ± 0.3) groups relative to the *E. coli* group (0.3 ± 0.46).

### 3.3. Plasma Concentrations of CRP, Procalcitonin, TNF-α, IL-1β, and IL-6

#### 3.3.1. Acute Phase Proteins (CRP and PCT)

**Figure 2:**
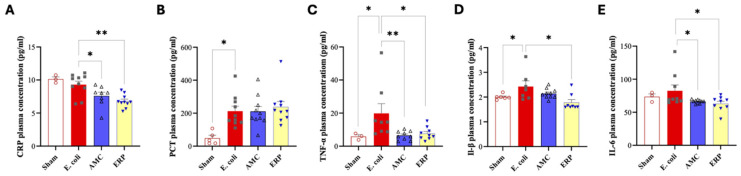
Comparison between each group regarding CRP (**A**), PCT (**B**), TNF-α (**C**), IL-1β (**D**) and IL-6 (**E**) plasma concentration. * *p* < 0.05. ** *p* < 0.01.

In the assessment of CRP levels (Figure 2A), the *E. coli* group (9.3 ± 1.66 pmol/mL) exhibited significantly higher plasma concentrations compared to both the AMC (7.59 ± 1.58 pmol/mL) and ERP (6.79 ± 0.99 pmol/mL) groups (*p* < 0.05). Regarding PCT levels (Figure 2B), a significant elevation was observed in the *E. coli* group (212.16 ± 95.5 pg/mL) relative to the Sham group (48.48 ± 38.38 pg/mL). PCT levels in the AMC and ERP groups remained elevated (210.93 ± 93.7 pg/mL and 237 ± 106.77 pg/mL, respectively) without significant divergence from the *E. coli* group.

#### 3.3.2. Pro-Inflammatory Cytokines (TNF-α, IL-1β, and IL-6)

Analysis of TNF-α concentrations (Figure 2C) showed a significant increase in the *E. coli* group (19.81± 16.74 pg/mL) compared to the Sham control (5.86 ± 1.95 pg/mL). Treatment with AMC (6.47 ± 2.74 pg/mL) or ERP (7.13 ± 3.8 pg/mL) resulted in a statistically significant reduction in TNF-α levels relative to the *E. coli* group (*p* < 0.05). Similarly, IL-1β levels (Figure 2D) were significantly higher in the *E. coli* group (2.43 ± 0.6 pg/mL) than in the Sham group (2 ± 0.1 pg/mL). A significant decrease was observed in the ERP group (1.78 ± 0.33 pg/mL; *p* < 0.05), while the AMC group (2.14 ± 0.2 pg/mL) showed a downward trend that did not reach significance. Finally, plasma IL-6 levels (Figure 2E) followed a similar pattern. While the increase from Sham (73.53 ± 7.31 pg/mL) to *E. coli* (82.6 ± 25.98 pg/mL) was not statistically significant, IL-6 concentrations were significantly reduced in both the AMC (66.3 ± 2.7 pg/mL) and ERP (62.9 ± 10.97 pg/mL) groups when compared to the *E. coli* group.

### 3.4. Measurement of Protein Expression

**Figure 3:**
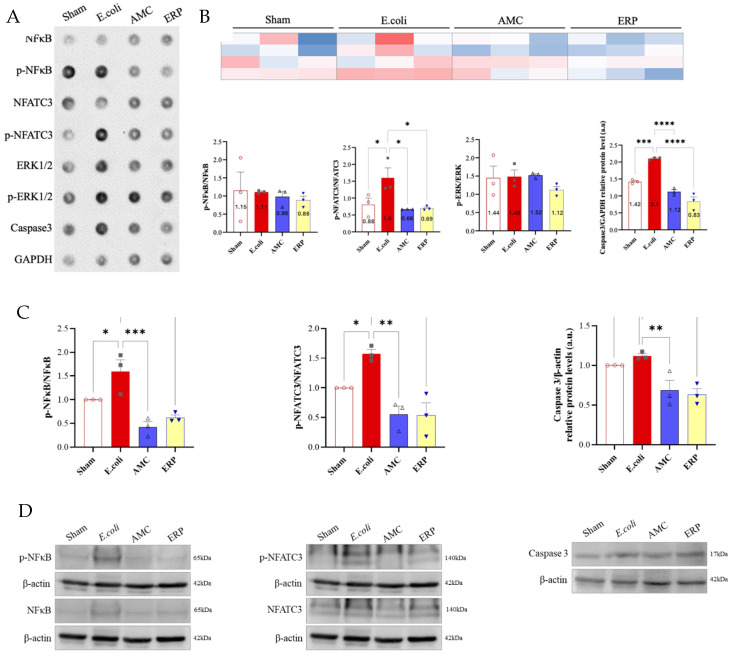
Proteomic study of the peritoneum. (**A**) Chemiluminescence dot study; (**B**) Densitometric quantification; (**C**) Statistical analysis of different protein expression pathways; (**D**) Western blot; Western blots and quantifications of NF-κB, phospho-NF-κB, NFATC3, phospho-NFATC3, ERK1/2, phospho-ERK1/2, caspase 3, GADPH and β-actin in rat peritoneal tissue. * *p* < 0.05. ** *p* < 0.01. *** *p* < 0.001. **** *p* < 0.0001.

**Figure 4:**
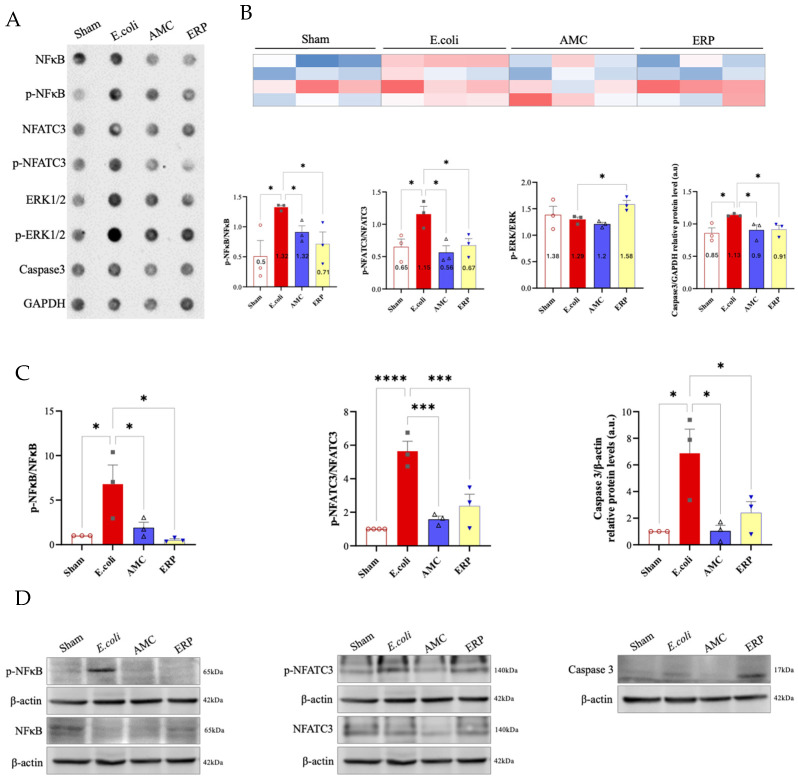
Proteomic study of the spleen. (**A**) Chemiluminescence dot study; (**B**) Densitometric quantification; (**C**) Statistical analysis of different protein expression pathways; (**D**) Western blots and quantifications of NF-κB, phospho-NF-κB, NFATC3, phospho-NFATC3, ERK1/2, phospho-ERK1/2, caspase 3, GADPH and β-actin in rat spleen tissue. * *p* < 0.05. *** *p* < 0.001 **** *p* < 0.0001.

Peritoneal and splenic expression of NF-κB, NFATc3, ERK1/2, and caspase-3 were evaluated (Figure 3 & Figure 4 respectively). In the peritoneum, pNFATc3/NFATc3 and caspase-3/GAPDH were significantly downregulated in the AMC and ERP groups compared to the *E. coli* control, while NF-κB and p-ERK/ERK remained largely unchanged. In the spleen, AMC and ERP treatments significantly reduced the expression of NF-κB, NFATc3, and caspase-3. Notably, splenic p-ERK/ERK levels were significantly higher in the ERP group than in the *E. coli* group.

### 3.5. Histopathology Study of Peritoneum, Spleen and Lung

**Figure 5:**
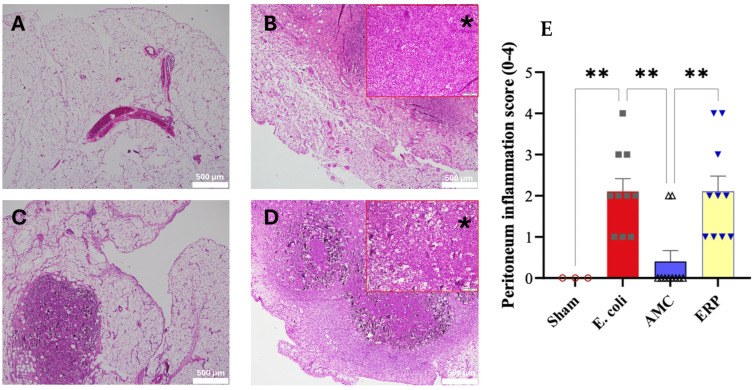
Microphotographs of representative histological sections of rat peritoneum with hematoxylin/eosin, scale bar: 500 μm; (**A**) Sham; (**B**) *E. coli* group (* Magnification: ×20); (**C**) AMC; (**D**): ERP (* Magnification: ×20; scale bar: 500 μm). (**E**) Comparison of the degree of inflammation in the peritoneum or score of inflammation in the peritoneum. ** *p* < 0.01.

Histological evaluation of the peritoneal sections revealed distinct morphological differences across the four experimental cohorts (Figure 5). The Sham group exhibited normal tissue architecture with no signs of pathological alteration. In contrast, both the *E. coli* and ERP groups displayed significant inflammatory infiltration characterized by neutrophilic polymorphonuclear cells and the presence of purulent exudate. Statistical comparison of the inflammatory scores indicated that the *E. coli* group experienced the most severe tissue inflammation, significantly exceeding all other cohorts. Notably, the AMC group demonstrated the lowest degree of inflammation among the treated groups, showing a statistically significant reduction in inflammatory markers compared to both the *E. coli* and ERP groups.

**Figure 6:**
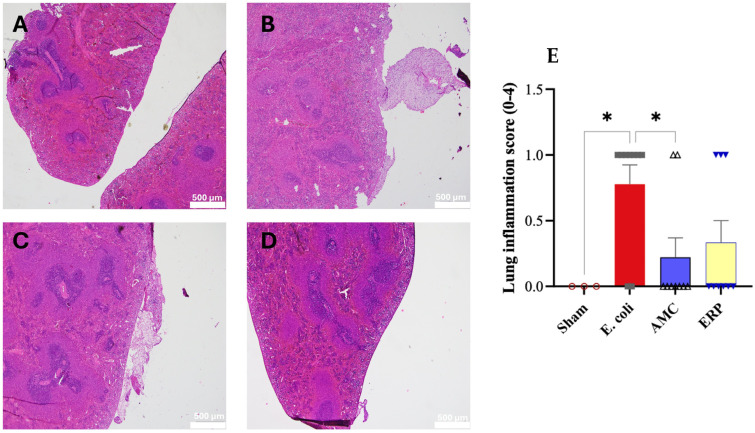
Microphotographs of representative histological sections of rat spleen with hematoxylin/eosin; scale bar: 500 μm; (**A**) Sham; (**B**) *E. coli*; (**C**) AMC; (**D**) ER; (**E**) Comparison of the score of inflammation in the peritoneum. * *p* < 0.05.

Histopathological examination of the spleen revealed a reactive phenotype characterized by neutrophilic aggregation (Figure 6). The degree of inflammation was significantly elevated in the *E. coli* group compared to the Sham group (*p* < 0.05). Conversely, both the AMC and ERP groups exhibited a downward trend in inflammatory infiltration, with the AMC group demonstrating the most pronounced reduction in splenic reaction.

**Figure 7:**
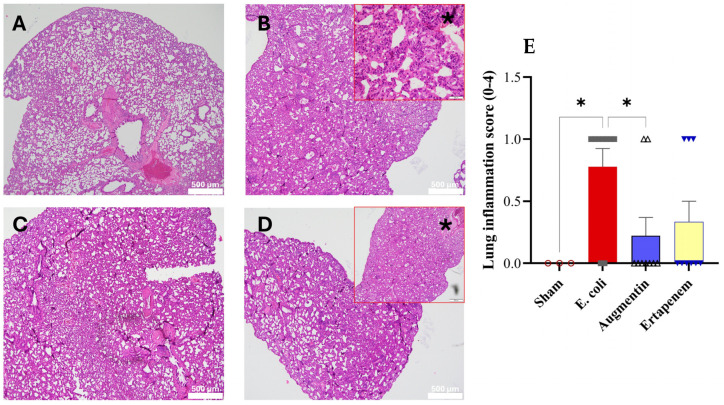
Microphotographs of representative histological sections of rat lung with hematoxylin/eosin at 500 μm scale; (**A**) Sham; (**B**) *E. coli* (* Magnification: ×40); (**C**) AMC; (**D**): ERP (* Magnification: ×40; scale bar: 500 μm). (**E**) Comparison of lung damage between groups. * *p* < 0.05.

Finally, our results showed lung involvement corresponded to 7/10 rats (70%) in the untreated E. coli group and 5/10 rats (50%) in both the AMC and ERP groups (Figure 7). A simple Fisher’s exact comparison between the *E. coli* group and each treated group did not reach statistical significance, consistent with the small group sizes and limited power of this exploratory study. Nonetheless, the absolute numbers indicate a numerically lower frequency of pulmonary involvement in the AMC and ERP groups compared with untreated animals, supporting the same directional trend observed for other inflammatory outcomes. The histopathology examination looked for endo-bronchial involvement and/or the presence of neutrophils in the lungs interstitium.

## 4. Discussion

Our study is the first to compare amoxicillin-clavulanic acid versus ertapenem in a rat model of ESBL-induced peritonitis, with all microbiological, physiological, and pathophysiological aspects; findings suggest potential similarity between AMC and ERP.

Our peritonitis model has been validated by Pechère et al. [13]. We prepared the bacterial solution with talc. Talc has pro-inflammatory properties on the serous membranes and is commonly used in medicine, primarily in pleurodesis [18]. In interpreting the inflammatory outcomes, it is important to acknowledge that intraperitoneal talc itself introduces a strong sterile inflammatory stimulus, independent of the bacterial challenge. While intraperitoneal talc is a validated adjuvant for inducing reproducible peritonitis, it is a potent inducer of sterile inflammation via inflammasome activation. Consequently, the inflammatory profiles observed likely reflect a synergistic effect between the mineral irritant and the microbial challenge, rather than a response to the pathogen in isolation. Consequently, part of the observed cytokine elevations and histological changes likely represent a combined effect of infection plus talc-induced inflammation. This should be considered when interpreting biomarker magnitudes. However, in our model, we observed statistically significant differences in both inflammatory cytokines and proteomic expression, independent of the effect of talc.

The comparison of positive microbiological cultures in the Sham, *E. coli*, AMP, and ERP groups showed a downward trend in the AMC and ERP groups compared to *E. coli* in the peritoneum and spleen, with a significant difference only in the peritoneum between *E. coli* and ERP. When a systemic infection, such as bacteremia, occurs, the spleen’s macrophages actively phagocytose and sequester the circulating bacteria. Therefore, finding bacteria in the spleen’s tissue is direct evidence that the bacteria have entered the systemic circulation and been filtered by this organ [19]. The positive microbiological results in samples taken from the spleen indicate bacteremia following the induction of peritonitis [20]. Although AMC and ERP showed similar downward trends in spleen culture positivity, the absence of a statistically significant difference between the two treatments does not imply equivalence or non-inferiority. Instead, this finding likely reflects the limited statistical power of this small exploratory study, which is insufficient to rule out clinically relevant differences in bacteremia clearance

To complete our study, we performed plasma assays of various inflammatory markers.

The profile of pro-inflammatory markers was more pronounced in the septic groups compared to Sham in the case of PCT, with TNF-α, IL-1β, and IL-6 indicating a persistence of the inflammatory state 7 days after sepsis induction, with a downward trend in the groups that received antibiotic therapy [21].

By analyzing CRP, we note that levels are between 5 and 10 pg/mL, with a statistically significant difference between *E. coli* and AMC, and *E. coli* and ERP. The Sham group has a higher CRP level than the rest. Current evidence of its potential utility as a biomarker for various diseases is validated, including cardiovascular, respiratory, hepatobiliary, gastrointestinal, pancreatic, renal, gynecological, andrological, dental, oral, otolaryngological, ophthalmological, dermatological, musculoskeletal, neurological, mental, splenic, thyroid conditions, as well as infections, suspected autoimmune diseases, and neoplasms [22]. However, the statistically significant difference between the two antibiotic groups and the *E. coli* group at day 8 validates our results.

As for PCT, no difference exists between the three septic groups. PCT is a useful marker for identifying bacterial peritonitis. Although PCT performs as well in patients on peritoneal dialysis as in those with cirrhosis or severe hepatitis, the use of a common cutoff value could further improve accuracy. Compared with CRP, PCT is superior for the diagnosis of bacterial peritonitis. However, it is important to note that PCT cannot yet be recommended as a “gold standard” test for peritonitis and must be interpreted in combination with other clinical, analytical, and/or microbiological data [23]. This lack of divergence in PCT suggests that the systemic inflammatory response to ESBL *E. coli* peritonitis was only partially modifiable by either antibiotic regimen under the dosing conditions used in this exploratory model. Therefore, the data do not support strong claims of systemic anti-inflammatory superiority for either AMC or ERP. Instead, the observed cytokine patterns should be interpreted as indicating directionally similar dampening of selected inflammatory pathways, rather than robust systemic resolution of sepsis-associated inflammation.

In bacterial infections, TNF-α, IL-1β, and IL-6 play crucial roles in the immune response. TNF-α and IL-1β are key proinflammatory cytokines involved in systemic bacterial infections, mediating organ damage, hypotension, and mortality, while IL-6 contributes to the production of acute-phase proteins and fever [24]. These cytokines are associated with disease severity and progression, with TNF-α and IL-6 levels correlating with infection severity [25]. Furthermore, IL-1 and TNF-α are essential to contain bacterial infections, as demonstrated in brain abscess models, where their absence led to increased mortality and bacterial load, highlighting their importance in the host antibacterial immune response. In our study, we have a decreasing trend in these 3 inflammatory markers with a statistically significant difference for IL-1β between the ERP and *E. coli* group, for TNF-α between the AMC and ERP groups on the one hand and *E. coli* on the other, and for IL-6 as well. This supports no major differences between AMC and ERP in the treatment of ESBL *E. coli* peritonitis.

In bacterial infections, the interaction between NF-κB and ERK1/2 is crucial for pathogenesis and the host immune response [26]. NF-κB activation is induced by bacterial components through Toll-like receptors, leading to the expression of pro-inflammatory genes [27]. ERK1/2, as an integral part of the MAPK pathway, plays a role in modulating cellular behavior and inducing inflammatory gene expression in response to infection [28]. These interactions highlight the complex mechanisms by which bacteria manipulate host cells and evade immune responses, highlighting the importance of understanding the roles of NF-κB and ERK1/2 in bacterial infections. Our results elucidate a downward trend in the AMC group compared to the *E. coli* group at the peritoneal level for NF-κB and p-ERK/ERK, as well as for pERK/ERK at the splenic level.

Caspases are a family of cysteine proteases that are central to both apoptosis and immune responses, acting as master regulators of these crucial cellular processes. They are synthesized as inactive precursors (procaspases) and are activated by proteolytic cleavage in response to specific signals [29]. The use of GAPDH as a loading control is particularly common in studies of apoptosis and cell death [30]. The interaction between caspase-3 and GADPH in bacterial infections represents a key mechanism by which host cells regulate apoptosis and the immune response. The cleavage of GADPH by caspase-3 modifies its functions and promotes the destruction of infected cells, thus limiting the spread of bacteria and enabling a more effective immune response [31,32]. We observed a statistically significant decrease in Caspase-3/GAPDH in the AMC and ERP groups compared to the *E. coli* group in both the peritoneum and spleen.

NFATc3 regulates the expression of cytokines and chemokines, which are essential for orchestrating the immune and inflammatory response [33]. NFATc3 has been shown to be essential for inducing TNF-α production, a key cytokine in the inflammatory response against bacterial infections. The role of NFAT signaling in fungal infections and in controlling the pattern recognition receptor NOD1 (nucleotide-binding and oligomerization domain-containing protein 1), which primarily detects invasive Gram-negative bacteria, is essential in the phagocytic functions of neutrophils [34]. Our results show a significant decrease in the AMC and ERP groups compared to the *E. coli* group.

Finally, the peritonitis in our study led to bacteremia with the development of sepsis. To study sepsis induced by peritonitis or bacteremia, large animal models were used, highlighting the importance of reliable animal models for understanding the pathophysiology of sepsis and developing new treatment strategies [35]. In the AMC group and at the peritoneal level, we observed a significant decrease in inflammation compared to the ERP group. This leads us to confirm that AMC has better peritoneal diffusion, as already confirmed in the study by Ricart et al. [36]. This same observation was found at the splenic and pulmonary levels, with no statistically significant difference. We found talc in the lungs of only one rat.

Several important limitations should be acknowledged when interpreting these findings. First, the study relied on a single ESBL-producing *E. coli* strain, which restricts generalizability given the genetic and phenotypic diversity among ESBL-producing Enterobacterales. Second, the work was conducted in one intraperitoneal infection model in otherwise healthy rats, which does not fully reproduce the complexity of human spontaneous bacterial peritonitis, where host factors such as cirrhosis, immune dysfunction, and altered peritoneal permeability strongly influence antibiotic performance. Third, the study employed a relatively small sample size typical of exploratory animal studies, and therefore lacked the statistical power needed to detect modest but clinically meaningful differences between treatment groups. For these reasons, the findings should be interpreted as hypothesis-generating preclinical evidence, indicating that AMC and ERP produced broadly similar trends across microbiological, inflammatory, proteomic, and histopathological endpoints in this controlled model. They do not establish therapeutic equivalence or non-inferiority, nor do they exclude the possibility of clinically relevant differences—particularly in bacteremia clearance or tissue penetration. Instead, the results support the rationale for conducting more robust follow-up work, including pharmacokinetic and pharmacodynamic studies, multi-strain evaluations, models incorporating clinically relevant comorbidities, and eventually well-powered clinical investigations to determine the settings in which AMC may serve as a narrower-spectrum alternative to carbapenems. Furthermore, if possible, a daily assessment of the rats’ cytokine profile would have strengthened our results.

Despite these limitations, the strength of our study lies in the first use of an animal model combining different aspects (microbiological, physiological, pathophysiological, and histopathology) to study the potential similarity of AMC with AMC in an induced peritonitis. Due to its originality, this study is a pilot study that has not yet been described in the literature.

## 5. Conclusions

Our model showed broadly comparable microbiological, biochemical, proteomic, and histopathological effects between AMC and ERP in the treatment of this type of peritonitis, with an effect on mortality and acute inflammation. This provides a basis for clinical studies evaluating the efficacy of narrower antibiotic therapy in the context of emerging antibiotic resistance. Our work opens new perspectives: conduct broader studies to transpose the ESBL peritonitis model in human peritonitis and to study the impact of amoxicillin-clavulanic acid on the selective pressure for bacterial resistance.

## Data Availability

The original contributions presented in this study are included in the article. Further inquiries can be directed to the corresponding author.
